# Modified Melody Valve Surgical Implantation in Atrioventricular Position in Four Children Under Two Years of Age

**DOI:** 10.1007/s00246-025-03972-9

**Published:** 2025-07-30

**Authors:** Andrés David Aranzazu-Ceballos, Luis Horacio Diaz, Rafael Correa Velásquez, Alberto Alejandro Quintero, Rafael Lince Varela, Guillermo José Aristizabal

**Affiliations:** 1 Pediatric Cardiology Fellow. Universidad Pontificia Bolivariana – Clínica Cardio VID, Medellín, Colombia; 2 Pediatric Interventional Cardiology Team. Clínica Cardio VID, Medellín, Colombia; 3 Pediatric Cardiothoracic Surgery Team. Clínica Cardio VID, Medellín, Colombia

**Keywords:** Mitral valve, Tricuspid valve, Cardiac catheterization, Melody valve

## Abstract

Mitral and tricuspid valve replacement options have traditionally been limited to mechanical valves, bioprostheses, and the mitral Ross procedure. In recent years, the Melody valve (Medtronic, Minneapolis, MN) has emerged as a viable alternative for infants and young children. We present four patients under two years of age who underwent surgical implantation of a modified Melody valve 22 mm in the atrioventricular position. The valve was placed in the mitral position in three patients and the tricuspid position in one. The procedure was performed with palliative intent in all patients, as no alternative surgical options were available. At the time of valve implantation, two patients were supported with venoarterial extracorporeal membrane oxygenation. At the time of this report, two patients remain alive, with a maximum follow-up of three years in one case following valve implantation. We describe the experience of using a modified Melody valve in the tricuspid or mitral position with palliative intent in four patients, supporting its viability as an option in children under two years of age with limited therapeutic alternatives.

**Supplementary Information:**

The online version contains supplementary material available at 10.1007/s00246-025-03972-9.

## Background

Mitral and tricuspid valve replacement options have traditionally been limited to mechanical valves, bioprostheses, and the mitral Ross procedure. Mechanical valves and bioprostheses are only available in sizes larger than 12 mm in diameter, restricting their use in neonates and infants with annular hypoplasia and leading to poor outcomes when implanted supra-annularly [1, 2]. Additionally, these prosthetic valves have fixed diameters, necessitating early reinterventions as they do not accommodate somatic growth. The Melody valve (Medtronic, Minneapolis, MN), a stent-mounted, valved bovine jugular vein graft, was initially designed for transcatheter pulmonary valve replacement in the right ventricular outflow tract [2–4]. Due to its ability to be implanted at a smaller diameter, with further ability to be expanded at a later age, it was adapted to be used for atrioventricular (AV) valve replacement via a surgical approach [2, 3]. This study aims to describe our institutional experience with the surgical implantation of a modified Melody valve in the AV position in small children.

## Materials and Methods

We present a case series of four children with severe AV valve disease who underwent surgical Melody valve implantation after a multidisciplinary evaluation (MDE). We retrospectively reviewed the charts of patients who received a Melody valve in the AV position at our institution between 2019 and February 2025. A detailed case-by-case description is provided. The institutional review board approved this study.

### Preoperative Evaluation

Echocardiography was used to obtain the anteroposterior and lateral dimensions of the annulus, determining both the feasibility of valve placement and the appropriate balloon size for implantation. A 2.5-cm mid-ventricle to mid-atrium clearance was required to accommodate the valve. The indications for replacement, implantation techniques, and short-term outcomes have been reported.

### Valve Preparation

**Fig. 1:**
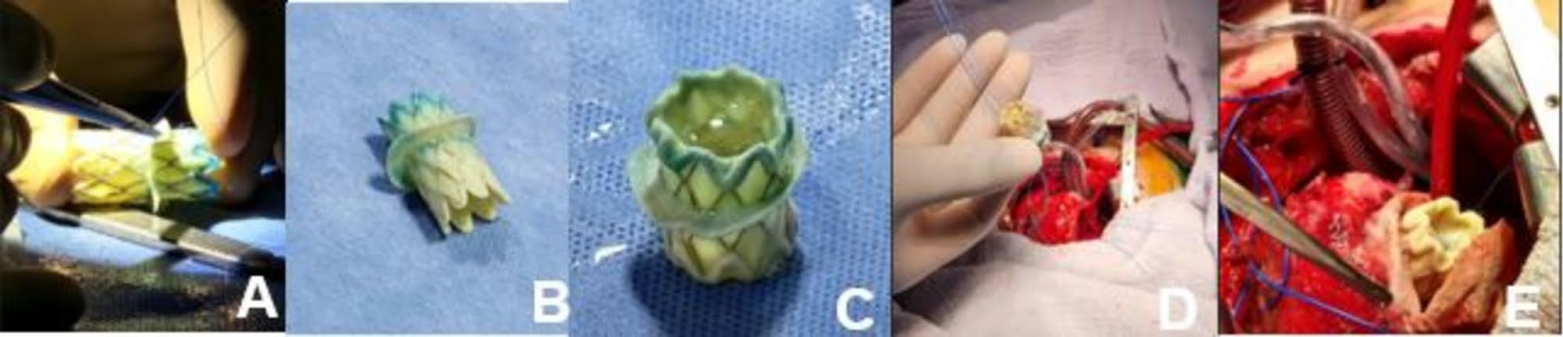
Implantation of the Melody valve. **A** Sheath with bovine pericardial patch. **B**, **C** Longitudinal reduction of the valve folds at its final portion. **D** Valve descent to the tricuspid plane. **E** Valve positioned at the tricuspid annulus

The Melody valve requires preparation before initiating cardiopulmonary bypass. It should be flushed with saline solution according to manufacturer specifications. Its longitudinal diameter can be adjusted by crimping the distal and proximal struts. A pericardial skirt is then sutured around the outer stent frame of the valve, midway between the base of the leaflets and the tips of the commissures, to facilitate suturing to the mitral annulus (Fig. 1A–C). Care must be taken to avoid damaging the valve leaflets; an intermittent infusion of saline solution can separate them from the sinuses. Finally, the valve must be compressed to the minimum diameter that a balloon catheter allows, ensuring the lumen is not completely obliterated.

### Surgical Technique

The AV valve is accessed via atriotomy, and the native valve and subvalvular apparatus are resected. The diameter of the native annulus is then measured using serial vascular dilators. The Melody valve is prepared by mounting it onto an angioplasty balloon. It is then positioned precisely within the measured annulus. Once correctly aligned, the balloon is inflated to expand the valve, which is subsequently secured to the valve annulus with a running circumferential suture.

For optimal expansion, the Melody valve should be inflated to 4–6 atmospheres using catheterization balloons no more than 1 mm larger than the measured annulus diameter (Z score 0). After implantation, the inflow segment of the valve must be inspected to ensure unobstructed pulmonary veins. The valve leaflets should also be assessed with saline to confirm proper coaptation and function (Fig. 1D, E).

Finally, an echocardiogram is performed to evaluate immediate valve function, rule out paravalvular leaks or obstruction of the outflow tract, and assess systemic/pulmonary vein patency and ventricular function.

### Postoperative Care

During the postoperative period, patients are initially anticoagulated with unfractionated heparin or enoxaparin until aspirin (5 mg/kg/day) and clopidogrel (<2 years—0.2 mg/kg/day and >2 years—1 mg/kg/day) can be initiated. All patients are followed by cardiology and cardiovascular surgery after discharge at 1, 3, and 6 months and thereafter at the specialist’s discretion.

## Results

### Case 1

This case involves a one-year-old female patient with a history of Down syndrome. At presentation, her weight was 9 kg and her height was 65 cm. Her underlying cardiac anatomy included a balanced complete AV septal defect (Rastelli type A) and a patent ductus arteriosus. At one month of age, she underwent ductus arteriosus ligation and pulmonary banding due to pulmonary overcirculation. At 9 months, she was admitted for surgical repair, which included pulmonary banding removal and complete AV septal defect correction using the two-patch technique. Intraoperatively, the right AV valve exhibited severe regurgitation. An attempt at valve plasty was unsuccessful, leading the surgical team to implant an EPIC SUPRA PLUS N19 bioprosthesis, which showed no leakage on the continence test. In the immediate postoperative period, the patient developed severe biventricular dysfunction, requiring central venoarterial extracorporeal membrane oxygenation (VA-ECMO) cannulation.

A Melody valve (22 mm) was successfully implanted in the right AV position as a palliative measure. The valve was expanded to a diameter of 14 mm using an XXL balloon inflated to 6 atmospheres and then secured to the valve annulus. Post-procedure, echocardiography confirmed adequate valve function with a 3-mmHg gradient.

Postoperatively, the patient remained dependent on VA-ECMO, with severe right ventricular dysfunction. Although right AV valve function was preserved, her condition remained unchanged. Given the lack of improvement, a joint decision was made with the family to withdraw VA-ECMO support, and the patient passed away the following day.

### Case 2

A two-year-old female patient. At presentation, her weight was 12 kg and her height was 69 cm. She had a history of Shone syndrome and, at seven days of life, had undergone successful repair of aortic coarctation and aortic arch reconstruction. At two years of age, she developed severe mitral regurgitation secondary to a perforation in the A2 segment of the anterior leaflet. She subsequently underwent mitral valve plasty and leaflet reconstruction. However, the intraoperative outcomes were poor, necessitating immediate support with central VA-ECMO. In the immediate postoperative period, echocardiography revealed persistent severe mitral valve regurgitation with significant hemodynamic compromise.

A 22-mm Melody valve was then successfully implanted in the mitral position as a palliative measure. The valve was expanded in the mitral position using a NuMED Canada Inc. Z-MED II™ 16 mm × 3 cm balloon, inflated to 6 atmospheres, and secured to the native mitral valve annulus. Transesophageal echocardiography immediately confirmed a mean gradient of 3 mmHg, with laminar flow and no obstruction of the left ventricular outflow tract (Fig. 3).

**Fig. 2:**
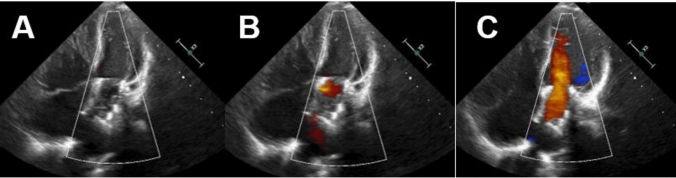
Follow-up echocardiography 2 years after the procedure. **A** Apical 4 C Melody valve. **B** Apical 4 C in diastole with Melody valve with adequate leaflet closure. **C** Apical 4 C with color Doppler without evidence of significant flow acceleration, with a mean valvular gradient of 5.5 mmHg

During three years of follow-up, the patient remained clinically stable, experiencing no complications or hospital readmissions and maintaining functional class I status. Serial echocardiographic evaluations consistently showed a normally functioning valve with a mean gradient of 5.5 mmHg (Fig. 2), without evidence of left atrial dilation or signs of pulmonary hypertension.

### Case 3

A 6-month-old female patient. At presentation, her weight was 6 kg and her height was 59 cm. She had a history of severe mitral valve stenosis, critical coarctation of the aorta, and hypoplasia of the aorta. At 5 days of life, the patient was taken to aortic arch reconstruction, mitral valvuloplasty, and a calibrated 5-mm atrial septal defect. At 5 months, the patient presented to the emergency room with signs of heart failure and evidence of severe mitral valve stenosis and insufficiency on echocardiogram, with a maximum gradient of 21 mmHg and a mean gradient of 12.5 mmHg. The patient was admitted to the intensive care unit. The patient underwent the palliative placement of a modified 22-mm Melody valve in the mitral position. The valve was expanded to 16 mm using a Z-MED II™ balloon, inflated to 6 atmospheres. Immediate transesophageal echocardiography confirmed a mean gradient of 3 mmHg with laminar flow.

During follow-up, the patient was stable, with significant improvement in the signs of heart failure, and without stenosis and mitral valve insufficiency, which allowed dismantling of management for heart failure. Two years after the procedure, the patient was readmitted to the intensive care unit with ventilatory failure due to respiratory syncytial virus infection, which is why he died 2 years and 8 months after the procedure.

### Case 4

An eight-month-old male patient, weighing 6.9 kg and 61 cm in height at presentation, had a complex cardiac history. His underlying conditions included Tetralogy of Fallot with mild pulmonary stenosis, a mitral supravalvular membrane, moderate mitral regurgitation, a subaortic membrane, and aortic root dilation. He underwent surgical correction, which involved infundibulectomy, closure of the interventricular communication with a pericardial patch, mitral valvuloplasty, and resection of the subaortic membrane.

In the early postoperative period, echocardiography revealed a double mitral lesion, characterized by stenosis (mean gradient of 9 mmHg) and severe regurgitation, associated with biventricular dysfunction (severe right and moderate left). The patient’s initial postoperative course was complicated, requiring ventilatory and inotropic support, with no significant improvement observed during the first two weeks. Consequently, a 22-mm Melody valve was successfully implanted in the mitral position as a palliative measure. The valve was positioned at the mitral annulus without complications and dilated using a 16 × 40 mm XXL balloon at 5 atmospheres.

However, in the immediate postoperative period, the patient experienced an unfavorable outcome due to stenosis between the newly implanted Melody valve and the anterior leaflet of the native valve, resulting in a mean gradient of 10 mmHg and a superior paravalvular leak. Reoperation with anterior leaflet resection and repositioning was therefore performed. Intraoperative evaluation confirmed valve competence, and subsequent echocardiography showed adequate valve function, without residual stenosis or regurgitation, with a mean gradient of 5 mmHg.

**Fig. 3:**
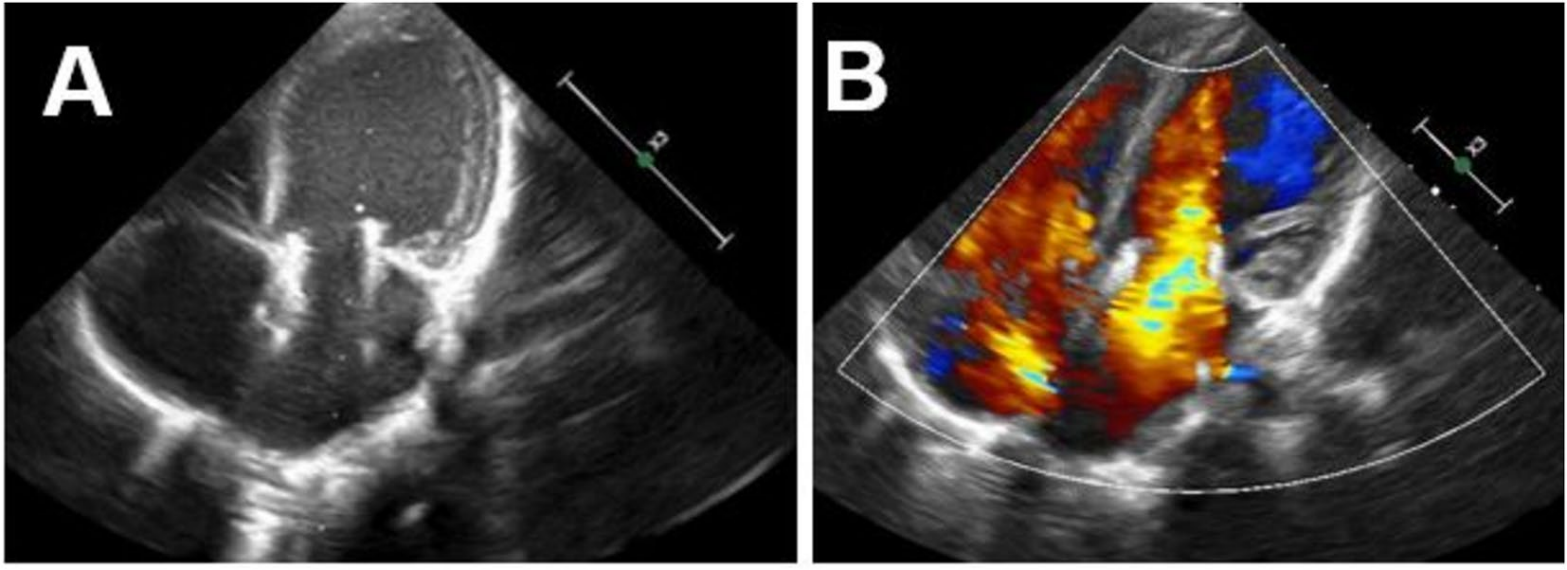
Follow-up echocardiography eight-month post-procedure. **A** Apical 4 C in systole with Melody valve with adequate opening. **B** Apical 4 C with color Doppler without evidence of significant flow acceleration, with a mean valvular gradient of 4.5 mmHg

The patient was discharged one month after the reoperation, with a Ross functional class II. He has been followed as an outpatient for eight-month post-procedure. Serial echocardiographic evaluations consistently showed a normally functioning valve with a mean gradient of 4.5 mmHg (Fig. 3), without evidence of left atrial dilation or signs of pulmonary hypertension.

## Discussion

Congenital mitral and tricuspid valve disease presents a significant challenge in pediatric congenital heart surgery. The recent adoption of balloon-expandable stent-mounted valves has introduced a promising alternative for atrioventricular valve implantation. This case series describes the surgical and hemodynamic experience of a high-complexity cardiovascular center following the implantation of a modified Melody valve in the atrioventricular position, demonstrating acceptable short- and medium-term outcomes (Table 1) [3–7].

**Table 1:** Summary of important clinical aspects of case series

Pt No.	Gender	Age (months)	Weight (kg)	Diagnosis	Previous procedures	22-mm Melody valve position - Implantation diameter	Postoperative Mean Gradient/Leak	Follow-up	Last status
1	Female	12	9	Rastelli A AV septal defect, severe right AV valve insufficiency	Correction of complete AV septal defect, implantation of EPIC SUPRA PLUS 19 biologic valve in tricuspid position	Tricuspid – 14 mm	3 mmHg/No Leak	2 weeks	Dead - refractory cardiogenic shock
2	Female	24	12	Shone syndrome	Aortic coarctation repair, end-to-end anastomosis, mitral annulus enlargement, and supravalvular membrane resection.	Mitral – 16 mm	3 mmHg/Mild paravalvular leak	3 years	Alive - Functional class l – Mean Gradient 5.5 mmHg – Small paravalvular leak
3	Female	6	6	Severe mitral stenosis, hypoplasia of the transverse aorta and coarctation of the aorta	Aortic arch reconstruction, mitral valvuloplasty	Mitral – 16 mm	3.5 mmHg/No Leak	2 years	Dead - Respiratory failure caused by respiratory syncytial virus
4	Male	8	6.9	Tetralogy of Fallot, mitral valve membrane, mitral regurgitation, aortic and aortic root dilatation	TOF correction, infundibulectomy, VSD closure, mitral valvuloplasty and subaortic membrane resection	Mitral – 16 mm	5 mmHg/No Leak	8 months	Alive - Functional class ll – Mean Gradient 4.5 mmHg

*AV* atrioventricular, *TOF* tetralogy of Fallot, *VSD* ventricular septal defect

The Melody valve offers several advantages over other commercially available prostheses. It can be implanted in neonates and infants with annular diameters smaller than 12 mm and has the potential for percutaneous expansion (up to 22 mm in diameter), accommodating somatic growth and potentially delaying the need for surgical repair or valve replacement. Additionally, advancements in valve technology and implantation techniques have enabled non-surgical replacement options [4].

Abdullah et al. [4] reported two successful cases of hybrid-modified Melody valve implantation in high-risk infants with favorable short-term outcomes. Quiñonez et al. [7] described 11 cases of modified Melody valve implantation at a mean age of 7 months, demonstrating acceptable short-term functional results; however, one patient died, and another required a permanent pacemaker.

Frigiola et al. [8] reported eight patients, ranging from 3 months to 6.2 years of age, who underwent Melody valve implantation in the mitral position. Two of these patients required ECMO support following traditional mitral valve repair and subsequently received a Melody valve as a rescue intervention, similar to the approach described in Case 1 of this series. Despite good valve function, both patients died, a finding consistent with the outcome of Case 1 in this report.

Chetan et al. [9] described 12 cases of Melody valve implantation in the atrioventricular position. Two cases were performed as a rescue intervention with mechanical support, but both patients died. In the remaining 10 cases, outcomes were compared with those of mechanical valve replacement, demonstrating equivalent transplant-free and reintervention-free survival rates, with a modest survival benefit at 1 and 3 years. Langer et al. [10] reported four cases of mitral valve replacement using a modified Melody valve implantation technique, achieving good results with no complications and favorable short-term hemodynamic outcomes.

A multicenter cohort study by Pluchinotta et al. [11] analyzed 68 patients who underwent Melody valve implantation in the mitral (59 patients) and tricuspid (9 patients) positions. In the immediate postoperative period, the valve demonstrated competence with low transvalvular gradients. However, during follow-up, 15 patients died, 3 required heart transplantation, and 19 underwent reintervention. At 12 months postoperatively, a cumulative incidence analysis indicated that 55% of patients remained free from death, heart transplantation, structural leaflet deterioration, or valve replacement. In our case series, Case 2 exhibited favorable evolution three years after the intervention.

Despite its advantages, Melody valve implantation in the atrioventricular position presents several challenges. First, the device has a length of approximately 3 cm, increasing the risk of protrusion into the left atrium or ventricle, which may lead to pulmonary vein stenosis or left ventricular outflow tract obstruction. Second, in situ valve dilation is performed to optimize the effective orifice area, ensure proper positioning, and minimize paravalvular leakage; however, this process carries a risk of injury to the coronary arteries and conduction system [2, 7]. Gilg et al. [12] identified factors associated with early valve failure, including age younger than 12 months, weight below 10 kg, and single-ventricle physiology.

While other transcatheter valve options—such as the Sapien and Myval valves—are available, their use in pediatric patients remains less common. These devices typically have a larger minimum diameter (≥20 mm) compared to the Melody valve, which generally restricts their application to older children [13, 14]. Moreover, limited availability of the Sapien and Myval valves in our clinical setting further constrains their use.

## Conclusion

This case series details the surgical implantation of a modified Melody valve in the AV position for palliative use in patients younger than two years of age with severe valvular disease. Our findings underscore the reproducibility of this intervention in patients whose age and size significantly limit conventional surgical options.

## Supplementary Information

Below is the link to the electronic supplementary material.

Supplementary file1 (PDF 135 KB)

## Data Availability

No datasets were generated or analysed during the current study.
