# Supplementary material for: Modified Melody Valve Surgical Implantation in Atrioventricular Position in Four Children Under Two Years of Age
Source: Pediatr Cardiol. 2025 Jul 30;47(4):1811–7. doi: 10.1007/s00246-025-03972-9 (PMC12946295; doi:10.1007/s00246-025-03972-9)
Supplement: Supplementary file 1 — Supplementary file1 (PDF 135 KB) [file 246_2025_3972_MOESM1_ESM.pdf]

Medellín, 17 de Febrero 2025

Investigador principal
Andrés David Aranzazu Ceballos
Universidad Pontificia Bolivariana
Medellín.

Asunto: Comunicación centro de investigaciones – Clínica Cardio VID

Proyecto: Implantación válvula Melody en posición valvular atrio ventricular: descripción de experiencia pediátrica

Cordial saludo,

Respetados investigadores:

El Centro de investigaciones de la Clínica CardioVID , aprobó la validación del proyecto de investigación titulado*: "*Implantación válvula Melody en posición valvular atrio ventricular: descripción de experiencia pediátrica". teniendo en cuenta que se trata de una validación externa posterior a un trabajo culminado, no es necesario el sometimiento al comité de ética de la clínica.

Recuerde que debe cumplir con los requisitos estipulados en el acuerdo de confidencialidad y carta de compromiso de la Clínica CardioVID.

Gracias por su atención,

Atentamente,

Sydney G

Sydney Goldfeder
Médica de Investigación y Coordinadora de Docencia Servicio
